# A Personalized and Interactive Web-Based Health Care Innovation to Advance the Quality of Life and Care of Patients With Heart Failure (ACQUIRE-HF): A Mixed Methods Feasibility Study

**DOI:** 10.2196/resprot.7110

**Published:** 2017-05-23

**Authors:** Susanne S Pedersen, Thomas Schmidt, Søren Jensen Skovbakke, Uffe Kock Wiil, Kenneth Egstrup, Kim G Smolderen, John A Spertus

**Affiliations:** ^1^ Department of Psychology University of Southern Denmark Odense Denmark; ^2^ Department of Cardiology Odense University Hospital Odense Denmark; ^3^ The Maersk Mc-Kinney Moller Institute University of Southern Denmark Odense Denmark; ^4^ Department of Medical Research Odense University Hospital Svendborg Denmark; ^5^ Saint Luke's Mid America Heart Institute and the University of Missouri Kansas City, MO United States

**Keywords:** feasibility, heart failure, patient-centered tools, mixed methods, Internet

## Abstract

**Background:**

Heart failure (HF) is a progressive, debilitating, and complex disease, and due to an increasing incidence and prevalence, it represents a global health and economic problem. Hence, there is an urgent need to evaluate alternative care modalities to current practice to safeguard a high level of care for this growing population.

**Objective:**

Our goal was to examine the feasibility of engaging patients to use patient-centered and personalized tools coupled with a Web-based, shared care and interactive platform in order to empower and enable them to live a better life with their disease.

**Methods:**

We used a mixed methods, single-center, pre-post design. Patients with HF and reduced left ventricular ejection fraction (n=26) were recruited from the outpatient HF clinic at Odense University Hospital (Svendborg Hospital), Denmark, between October 2015 and March 2016. Patients were asked to monitor their health status via the platform using the standardized, disease-specific measure, the Kansas City Cardiomyopathy Questionnaire (KCCQ), and to register their weight. A subset of patients and nursing staff were interviewed after 3-month follow-up about their experiences with the platform.

**Results:**

Overall, patients experienced improvement in patient-reported health status but deterioration in self-care behavior between baseline and 3-month follow-up. The mean score reflecting patient expectations toward use prior to start of the study was lower (16 [SD 5]) than their actual experiences with use of the platform (21 [SD 5]) after 3-month follow-up. Of all patients, 19 completed both a baseline and follow-up KCCQ. A total of 9 experienced deterioration in their health status (range from 3-34 points), while 10 experienced an improvement (range from 1-23 points). The qualitative data indicated that the majority of patients found the registration and monitoring on the platform useful. Both nursing staff and patients indicated that such monitoring could be a useful tool to engage and empower patients, in particular when patients are just diagnosed with HF.

**Conclusions:**

The use of patient tracking and monitoring of health status in HF using a standardized and validated measure seems feasible and may lead to insights that will help educate, empower, and engage patients more in their own disease management, although it is not suitable for all patients. Nursing staff found the patient-centered tool beneficial as a communication tool with patients but were more reticent with respect to using it as a replacement for the personal contact in the outpatient clinic.

## Introduction

Heart failure (HF) is the end stage of most heart diseases and a progressive, debilitating, and complex clinical syndrome characterized by dyspnea, edema, pulmonary congestion, decompensation, fatigue, impairments to daily functioning and quality of life [1,2], and risk of frequent hospitalizations and death [3]. Due to an increasing incidence and prevalence, which is expected to continue the next 20 years [4,5], HF represents a global health and economic problem at a time when health care systems worldwide are challenged. Hence, there is an urgent need to evaluate alternative care modalities to current practice to safeguard a high level of care for this growing population.

To date, HF care and disease management modalities that have been more clinically driven and relied on a mixture of telemonitoring, clinician monitoring and rating of symptoms based on more biometric measures and clinician-initiated contact have shown mixed results [6,7]. In addition, there is no close relationship between the vast majority of physicians’ traditional objective indicators of HF severity (eg, New York Heart Association [NYHA] functional class, electrographic, and hemodynamic parameters) and patients’ own assessment of their health status and quality of life [8]. By contrast, patients’ rating of their own health predicts mortality and hospital readmissions in HF independent of somatic disease indicators and traditional biomedical risk factors [9-11]. However, no proxy measure for patient-rated health status can be captured from patient medical records nor is standard screening for patient-reported health status part of clinical cardiology practice in Denmark today.

Thus, patient-rated health status could be used with advantage as one of the patient-centered tools in clinical practice to monitor patients’ health status, which may allow the timely detection of clinical deterioration in their HF condition and enable treatment recommendations to be tailored to individual patient needs and preferences [12-14]. This represents a systems review of the body beyond what traditional biometric measures can offer and is sustainable over time in contrast to technological solutions that may rapidly become outdated and replaced. A one-size fits all approach [6,15] and the absence of a patient-centered approach are likely to have contributed to the failure of available HF care and disease management modalities [16,17]. In addition, a recent study advocates a paradigm shift that moves away from “...individual blame toward an empowerment and systems approach that considers the big picture” [17].

Hence, we designed the ACQUIRE-HF study (a personalized and interactive Web-based health care innovation to advance the quality of life and care of patients with heart failure) to examine the feasibility of engaging patients to use patient-centered and personalized tools coupled with a Web-based, shared care, and interactive platform in order to empower and enable them to live a better life with their disease. The feasibility study is a precursor to a large randomized controlled trial that will open up for more features on the platform and include a psychological intervention for the subset of patients who score high on anxiety and depression. The results presented in this paper include both quantitative and qualitative data on experiences with the platform and the intervention both from the perspective of patients and nursing staff.

## Methods

### Study Design and Population

We used a mixed methods, single-center, pre-post design. Patients with HF (n=26) were recruited from the outpatient HF clinic at Odense University Hospital (Svendborg Hospital), Denmark, in the period between October 2015 and March 2016 as a convenience sample. Patients were asked to complete purpose-designed, standardized, and validated questionnaires at baseline and at 3-month follow-up. Nursing staff (n=6) from the outpatient HF clinic was involved in the study.

The qualitative study consisted of (1) observations during the workshop and training course of the nursing staff in use of the platform, which was provided by CGI Denmark, (2) observations during the workshop and training of 5 of the 26 patients on how to use the platform, which was provided by nursing staff, (3) semistructured telephone or face-to-face interviews with 10 patients after they had used the platform for 3 months (patients were interviewed toward the end of January 2016), and (4) focus group interviews with 3 of the 6 nurses, which were conducted at a time when the majority of the 26 patients had been included in the study.

The Health Innovation Centre of Southern Denmark was responsible for all observations and interviews. Nurses provided continuous feedback, and a midway evaluation was conducted with nursing staff, CGI Denmark, and the research team from the University of Southern Denmark.

### Ethics

We submitted the study protocol to the Regional Committees on Health Research Ethics for Southern Denmark. According to Danish law on ethics related to health research (Law 593 of June 14, 2011), ethical committee approval is not required for this kind of study. The study was performed according to the Helsinki Declaration. Permission was also sought and granted from the Danish Data Protection Agency under the umbrella agreement of the University of Southern Denmark (2015-57-0008).

### Platform Used for the Study

We used CGI’s modular and cloud-based CommunityCare360 (CC360) platform in the study, with patients and nursing staff having access to the platform and its tools via a Web interface on a tablet, smartphone, or computer. CC360 makes it possible for patients, health care professionals, and other stakeholders to access, monitor, and update personal health data. It allows for (1) integration of information from various sources including health technology (eg, weight scale) that patients may use, electronic health record (EHR), labs, imaging, and prescribing; (2) relatives to gain access to patient data provided that patients give their permission; (3) patients to write messages to the HF team; (4) patients and caregivers and health care professionals to engage in video dialogs; and (5) setting targets for patients’ medication, which may serve as a reminder to patients and health care professionals if targets are not met. Manuals are available to support use for patients, clinicians, and other health care professionals. The platform has a health care classification toolkit (SNOMED, *International Classification of Diseases, 10*^th^*Edition* [ICD-10], ICD-10 procedure codes [ICD-PC], Nomenclature for Properties and Units [NPU] result codes, etc) and facilitates easy device integration through the Sensor engine, which supports the Continua Alliance standard. The platform gives instant feedback in a red-amber-green color state methodology, clearly showing the patient if the measurement is okay (green), if the patient should consult with a doctor (amber), or if the patient must consult with a doctor (red). This acts as a guideline both to patients and physicians to take action in the case of amber and red alerts.

**Figure 1:**
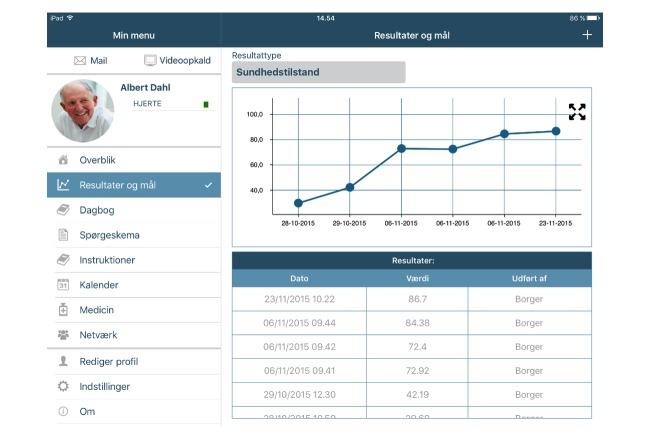
CommunityCare360 interface as seen by patients.

We used only 2 features on the platform for the feasibility study: patient registration of their weight and completion of a health status measure, allowing for patients to monitor their weight and health status over time. We started out with only these 2 features as HF patients tend to be somewhat older and not necessarily used to using technology [18]. We chose these 2 specific features as a try out, as weight monitoring is an essential part of HF management because an increase in weight can be a sign of congestion and decompensation [19] and patient-reported health status has been shown to be an independent predictor of rehospitalization and mortality [9]. Figure 1 presents the interface of the CC360 platform as seen by patients in the feasibility study.

### Measures

Information on baseline demographic and clinical characteristics was either captured from purpose-designed questions in the questionnaire or from the patient medical records. Data on patient compliance with reporting their weight and health status on the CC360 was available from the platform. Patients were asked to complete the following measures pretest (ie, at baseline) and posttest (ie, at 3-month follow-up).

#### Patient Expectations Toward and Experiences With the Platform

We developed a 11-item purpose-designed questionnaire to tap into patient expectations and experiences with the platform, with choice of items inspired by the standardized and validated Expectations Towards ICD Therapy (EXPECT-ICD) questionnaire [20]. We used the same items to tap into patient expectations toward use of the platform pretest and their experiences posttest, with the only difference in the wording being the use of the present versus past tense (eg, pretest: “Do you expect that use of the platform will make you feel more safe?” versus posttest: “Do you feel that the platform has led to you feeling more safe?”). The questionnaire contained both negatively and positively worded items. Items were rated on a 5-point Likert scale from 0 (completely disagree) to 4 (completely agree). Negatively worded items were recoded prior to calculating a total score. The score range of the scale is 0 to 44, with 44 representing the highest level of expectations and best possible experience with the platform.

#### Kansas City Cardiomyopathy Questionnaire

The Kansas City Cardiomyopathy Questionnaire (KCCQ) is a disease-specific, validated, patient self-report international standard used to quantify patient experiences with HF [21] that can capture clinical changes in patient condition and predict hospitalization and mortality [9,22] independent of N-terminal pro-brain natriuretic peptide, a biometric measure predictive of HF progression and mortality [23]. We used the 12-item version to reduce patient burden—an abbreviated version of the 23-item scale [24]—with equivalent validity and reliability [25]. Via an algorithm, KCCQ scores are converted to a score from 0 to 100, with 100 representing the best possible health status.

#### European Heart Failure Self-Care Behavior Scale

The European Heart Failure Self-Care Behavior (EHFScB) scale is a 9-item standardized and validated questionnaire that assesses patient opinions on their ability to manage their HF (eg, “I take my medication as prescribed”), with items answered on a 5-point Likert scale from 1 (completely agree) to 5 (completely disagree) with a score range of 9 to 45, with 9 representing best possible self-care behavior [26].

### Study Procedure

Nurses from the outpatient HF clinic approached patients for study participation and provided them with written and oral information about the study. After signing an informed consent form, patients were asked to complete the baseline questionnaire. Patients were also asked if they would be willing to be interviewed about their experiences with use of the platform after 3 months. Nurses from the hospital set up patients on the platform, and patients received a log-in and password to gain access. Nurses also trained all patients how to use the platform and patients received a user manual for CC360 to take home to facilitate use. Patients could contact the outpatient clinic if they encountered any problems with use of the platform (eg, problems with logging in, questions related to the use of the platform) with the nurses being the first point of contact for technical support. CGI Denmark provided back-up support to the nursing staff. Patients were also informed that use of the platform was not a substitute for usual care and that they should contact the clinic if they felt unwell and experienced deterioration in their condition or contact emergency services outside office hours.

### Intervention

As part of the intervention, patients were asked to do the following:

- Weigh themselves every day in the morning on their own scale and enter the weight into the platform. The rationale was that both the patient and the nursing staff in the outpatient clinic could monitor the patient’s weight and that patients might gain more insight into their weight fluctuations.
- Complete the KCCQ every 2 weeks during the 3-month study period as an indication of their health status. Scores were plotted in a graph that was visible both to patients and the nursing staff. Patients were instructed how to interpret their score and could also see the evolution in their health status over time.

The nursing staff had the following responsibilities:

- Call patients 1 week after study inclusion to ensure that patients could log in to and use the platform and were able to comply with the tasks (ie, entering and monitoring their weight and health status) as indicated above.
- Check every time patients had completed the health status measure (KCCQ). A reduction in patient score by 20% or more as compared to their baseline value instigated a red alert, a reduction between 10% and 20% an amber alert, and a reduction of 10% or less a green alert. These alerts appeared in the nursing staff’s module on CC360, which required them to contact the patient to discuss if the patient needed to be seen in the clinic.
- If patients would forget to enter their weight or to complete the KCCQ, a red light would appear on the nursing staff’s module on CC360. They would then be required to contact the patient to remind the patient to complete the measures.

### Data Analysis

Results related to the quantitative data are reported as frequencies with percentages or as means and standard deviation. Pearson correlation coefficients were calculated to examine the relationship between continuous measures. Data were analyzed using RStudio version 0.99 (RStudio Inc) and SPSS Statistics for Macintosh version 22.0 (IBM Corp). The qualitative data were analyzed using thematic analysis, with the aim of grouping data and finding patterns that give insight into the user experiences with the platform [27].

## Results

### Quantitative Data

Baseline characteristics of the patient sample are presented in Table 1. The mean age was 67 (SD 11) years, and the majority of patients were men with an NYHA functional class I-II (ie, asymptomatic or mild symptomatic HF).

### Study Attrition

The 7 patients who did not complete the posttest questionnaire had a lower baseline mean score on expectations toward the platform (15 [SD 4] vs 16 [SD 6]), a better self-care behavior score (13 [SD 3] vs 18 [SD 5]), and a lower health status score (61 [SD 21] vs 69 [SD 21]) as compared to the 19 patients who completed both the pre- and posttest questionnaires.

**Table 1:** Baseline characteristics of the patient cohort (n=26).

		Total
Age (years), mean (SD)		67 (11)
Men, n (%)		21 (81)
Married/have a partner, n (%)		20 (77)
Lower educational level (<14 years), n (%)		15 (58)
Employed, n (%)		7 (27)
**NYHA class^a^(severity of heart failure), n (%)**		
	I-II	24 (92)
	III-IV	2 (8)
**Comorbidities (based on self-report), n (%)**		
	Stroke	4 (15)
	Diabetes	2 (12)
	Aneurism	1 (4)
	Liver disease	0 (0)
	Kidney disease	0 (0)
	Claudicatio intermittens	0 (0)
	COPD^b^	3 (12)
	Ulcer	1 (4)
	Cancer during last 5 years	1 (4)
	Other	5 (19)

^a^NYHA: New York Heart Association functional class (III-IV: most severe heart failure).

^b^COPD: chronic obstructive pulmonary disease.

**Table 2:** Pre- and posttest scores on the questionnaires.

	Baseline pretest		3-month follow-up posttest	
	Valid cases (n)	Mean (SD)	Valid cases (n)	Mean (SD)
Expectations: use of the platform^a^	25	16 (5)	—	—
Experiences: use of the platform^a^	—	—	18	21 (5)
Health status: KCCQ^b^	26	62 (21)	19	68 (15)
Self-care behavior: EHFScB^c^	26	17 (5)	19	18 (6)

^a^The same items were used to tap into patient expectations toward use of the platform pretest and their experiences posttest with the only difference in the wording being the use of the present versus past tense (see the Methods section). Score range was 0 to 44 (44 = highest level of expectations and best possible experience).

^b^Kansas City Cardiomyopathy Questionnaire (KCCQ) score range 0 to 100 (100 = best possible health status).

^c^European Heart Failure Self-Care Behavior (HFScB) score range 9 to 45 (9 = best possible self-care behavior).

### Patient Expectations and Experiences and Actual Use of the Platform

Patient scores with respect to expectations toward and experiences with the platform are displayed in Table 2. Patient actual use of the platform, as indicated by the number of times that patients were logged on to the platform, varied considerably from 2 to 210 times during the 3-month follow-up period, with total number of log-ins being 2968 and the mean being 114 (SD 72) times (median 140, interquartile range 131). A total of 3 patients never logged on to the platform. Patients with higher expectations toward use of the platform at baseline reported a higher score with respect to their experiences after 3 months (Pearson *r*=.41; *P*=.10), with expectations toward use of the platform accounting for 17% of the variance in patient experiences with the platform.

**Table 3:** Pre-, posttest, and change health status Kansas City Cardiomyopathy Questionnaire scores and weight entries on the platform for individual patients during 3-month follow-up.

Patient ID	KCCQ^a^ score baseline	KCCQ score 3-month follow-up	KCCQ change score	KCCQ platform entries (count)^b^	Weight platform entries (count)^c^
1	85	—	—	0	1
2	72	63	–9	4	84
3	76	—	—	0	0
4	87	92	5	6	65
5	46	51	5	6	84
6	31	—	—	2	14
7	69	—	—	5	77
8	37	57	20	6	84
9	43	—	—	1	1
10	98	64	–34	5	82
11	51	74	23	5	85
12	81	75	–6	6	78
13	48	50	2	6	75
14	45	60	15	6	76
15	72	62	–10	6	64
16	93	90	–3	5	85
17	76	—	—	4	51
18	46	—	—	6	85
19	74	52	–22	6	84
20	96	92	–4	2	21
21	100	88	–12	1	1
22	65	57	–8	6	83
23	52	55	3	6	78
24	58	66	8	4	86
25	84	85	1	5	84
26	50	58	8	6	79

^a^KCCQ: Kansas City Cardiomyopathy Questionnaire.

^b^Possible KCCQ entries during 3-month follow-up period = 6.

^c^Possible weight entries 3-month follow-up period = 90.

### Changes in Health Status and Self-Care Behavior and Weight Entries

Table 2 presents the mean and SD pre- and posttest scores on the questionnaires. Overall, patients experienced improvement in patient-reported health status but deterioration in self-care behavior between baseline and 3-month follow-up. The mean score reflecting patient experiences with use of the platform was higher than their expectations toward use prior to start of the study.

Pre-, posttest, and change health status scores and number of weight entries for individual patients are presented in Table 3. Of the 19 patients who completed the baseline and follow-up KCCQ, 9 experienced deterioration in their health status score (range 3-34), while 10 experienced an improvement (range 1-23). Of 26 patients, 4 patients entered their weight once or not at all, while 19 patients entered their weight 64 times or more (range 0-86).

### Qualitative Interview Data

Based on interview data and observations, patient and nursing staff evaluations of the platform are summarized below according to specific topics.

#### Suitability of an Information Technology Solution to Patients With Heart Failure as Target Group

Given the demographics of patients with HF, one of the obvious questions to ask is whether an information technology (IT) solution as presented in ACQUIRE-HF is feasible. HF patients are typically older and do not necessarily have a lot of experience with such solutions or the confidence or the energy to engage in digitization. Our experiences show that patients who are unfamiliar with iPads and touch screens were significantly challenged already when having to log on to the platform. These challenges included scrolling down too quickly, not knowing how hard to touch the screen, etc. The observer noticed in some cases an increasing sense of insecurity and decreasing motivation, in particular in patients who borrowed an iPad for the project (they were not used to using it). For patients using their computer, it was much easier for them to understand and use the platform irrespective of their IT experience and user level.

Technological problems and nursing staff uncertainty when introducing and teaching patients about the platform—particularly in the beginning of the study—increased patient insecurity and discouragement with respect to participating. Thus, it is important that training of nursing staff in use of the platform occurs close to study start so they still have benefits from the training. However, experience is also built up over time. Hence, more intensive use of the system will likely create more confidence when problems do occur about how to resolve them.

Patients with very limited IT experience need a more thorough introduction to the use of computer/tablet, and they have a need for troubleshooting particularly in the early stage as these patients might be more prone to dropping out.

I could not figure it out—I kept trying but I couldn’t—but it is difficult. Nobody had the time to come and help me. They are all too busy—my children and grandchildren, they go to work and school. Then the nurse told me I could hand it in [the tablet]. ...I never really got into it. I didn’t really feel like familiarizing myself with it—but they also did not spend a lot of time showing me. Perhaps had it been my grandchildren I would have been more motivated to try it.

Patient, 83-year-old female

#### Patient User Manual on the Platform

Patients also indicated that more information is warranted on use of the platform and not just on the technical aspects. There are several concepts that they would have liked explained in more detail: “What is a reference value?” “What does health status refer to?” “What do the numbers mean?” “What can you use the diary for—how does that help me?”

I spent a lot of time reading the manual. I don’t think that everybody can understand it. It should probably be more detailed and informative both with respect to text and pictures.

Patient, 61-year-old male

#### User Friendliness and Customization of the Platform to the Individual Patient’s User Level

Patient experiences with computers and technology vary considerably. Hence, there is a need to customize the platform such that patients who have less experience start with a very simple set-up, while more experienced and curious users might be able to use and have benefit from more features on the platform.

We also have patients who would like to receive it digitally.

Nurse

#### Reminders via the Platform

Patients agree that it is important that the platform can send reminders to patients when they need to complete the health status measure and report their weight or if they have forgotten to do so. However, they prefer that the user is able to decide whether the reminder system should be switched on or off.

#### Completion and Monitoring of Patient-Reported Health Status via the Platform

The majority of interviewed patients found the questions strange and criticized that they had to answer the same questions every time.

The questionnaires are too general. Who has come up with these questions? Is it at all people who know about patients with heart disease?

Patient, 64-year-old male

Despite this criticism, other patients found the questionnaires valuable because they made them reflect and think things through. Nursing staff feels that completion of the questionnaires 2 times a month is too frequent and that once a month might increase motivation.

The principles behind the questionnaires are fine—as a matter of fact I think it is nice.

Patient, 82-year-old male

Patients voice a preference for being able to have a complete overview and see the evolution in their weight and health status scores over time.

I would like to see the entire month. I use the graph, as I have experienced a drastic weight loss in the middle of the period.

Patient, 70-year-old male

#### Patients’ Perceived Value of the Platform

Patients have different views of the platform and its potential usefulness. Generally, as the interview progresses patients attribute more and more value to the rationale behind the platform as they voice their experiences during the last 3 months. However, there are patients at both ends of the acceptance and value continuum. Overall, patients find the rationale and the idea behind the platform good, but the questions as formulated in the questionnaires are considered a major drawback. A few patients—3 out of 10 interviewed—do not find the platform useful nor do they believe that it could be useful to them in the future.

#### Increased Insight and Empowerment

Of the 10 patients interviewed, 7 patients either experience or believe that in the future the platform will help them better understand themselves, their body, and their disease and increase empowerment. They find both the insight but also the responsibility valuable.

I have been staying at home for many years. It was nice to have the platform to be able to monitor my own health. You gain knowledge about your disease and get a feeling that you are more involved in the process.

Patient, 82-year-old male

It is a nice tool that makes it possible to see how hard it has been. It is food for thought. It is nice to gain this insight. I become aware of things when I answer the questions—I can follow my own developments.

Patient, 61-year-old male

I have become aware of what I gain from weighing myself daily with respect to fluid retention. It is nice to know that you can follow your own disease in such a simple way. But you can become worried! But it gives a sense of security that you know what is going on—this way you can quickly relate your condition to your weight.

Patient, 60-year-old male

#### Better Communication

Patients feel that the platform is valuable as a communication tool and that the questions support their dialog with the nursing staff. In addition, it makes them remember important aspects related to their condition and health.

We can talk better now. Because you can refer to the questions—and it makes it a more equal dialog with the nursing staff.

Patient, 82-year-old male

#### Value for the Nursing Staff

The nursing staff feels that the platform is primarily valuable to patients, as the staff members already have dialogs with patients and their own way of monitoring patients’ weight and methods of screening. When asked about the potential of the platform in the future for themselves and patients, they feel that they learn something new about the patients and gain new (and more honest) insight into patients’ conditions. They experience that use of the KCCQ tells something new about patients’ health status and also that it provides them with different information than what patients tell them when they are seen in the outpatient clinic.

It is about how they deal with and accept their disease. We can have an opinion about how they feel, but here the questionnaire data can show a different and more true picture.

Nurse

Generally, the nursing staff felt that the platform facilitates a more equal dialog between patients and staff but that it cannot replace the personal contact in the outpatient clinic.

#### Implementation of the Platform in Clinical Practice

It is paramount that the expectations of patients and nursing staff are aligned and that patients are aware that they need to take an active role and act on their own scores when clinically relevant changes occur. The majority of patients express that they thought there would have been more dialog and follow-up based on their scores. They feel that they did not know what was going on at the other end (the nurses’ role).

I thought that there would have been more dialog between me and the hospital, such as videoconferencing, et cetera. I wrote a remark on the platform in the comment field, but nobody saw it. So it felt like somewhat of a dead end.

Patient, 60-year-old male

Nurses are concerned that patients might have inexpedient expectations that nurses act as contact person.

It is important to be prudent and not to cultivate the idea that we have to be part of their network.

Nurse

#### Timing for Introducing the Platform

Both patients and nursing staff feel that the platform could be of considerable value to newly diagnosed HF patients as a tool to develop good routines from the beginning and to gain knowledge of oneself, one’s disease, and the evolution of the disease.

It would be good when you are first diagnosed to learn about your disease.

Patient, 82-year-old male

It would give us important information about their level of functioning if the platform was used as part of the introduction to the outpatient clinic. Instead of us having to ask them, they could complete the questionnaire via the platform. Then patients would also have time to think about how they actually feel before they respond. Then we would have something to go on—then we know what the problem is without having to spend time on asking these questions... But it should probably not be digital—an IT platform is a bit overwhelming for most of our patients in the beginning.

Nurse

As such, nursing staff emphasizes that the platform should be viewed as a tool to support dialog, not as a replacement for contact with the outpatient clinic, and also a tool that patients can take with them and use once they are no longer seen in the clinic.

Based on the interview data and observations, recommendations for use of a platform and patient-centered tools in studies like ACQUIRE-HF are provided in Textbox 4.

**Recommendations based on the results of the feasibility study.**

Use of the platform and monitoring of symptoms:

- Should be customized to the individual patient’s user level and preferences if feasible
- Should facilitate that patients can use the technology that they are familiar with
- Is not a one-size fits all solution
- Is not a replacement for clinical practice
- Could be a useful tool for newly diagnosed patients
- Is a useful communication tool
- Gives patients a more equal relationship with nursing staff
- Provide nursing staff with new, additional and more honest information about patients
- Might induce anxiety in some patients

Practical and logistic issues:

- Alignment of patient and nursing staff expectations is paramount
- Patients prefer to see total overview and evolution in scores
- Reexamination of questionnaire use for health status monitoring
- Train nursing staff in use of the platform close to recruitment and allow a few test cases to increase familiarization

## Discussion

### Principal Findings

The ACQUIRE-HF study was designed to examine the feasibility of using a Web-based platform combined with patient-centered tools to empower and engage patients as more coactive partners in their own disease management. Despite concerns that such a solution might not be feasible to use in the HF population due to their higher age and risk of being inexperienced and challenged with respect to the use of a technology-based intervention, our results based on both quantitative and qualitative data show that such a solution is feasible but also that it is not a one-size fits all solution, with some patients (albeit a minority) never logging on to the platform or only using the platform a few times. Patients were not explicitly singled out for interviews if they did not enter their weight or use the platform. When patients were recruited for the study they were asked whether they would be willing to be contacted later for an interview. However, as indicated in one of the quotes included in the paper, insufficient coaching and familiarity with the platform could be one reason why this subset of patients did not engage with the platform. This was also supported in a recent study on extra device monitoring in patients with HF [28].

In this study, both patients and nursing staff recommended that patient health status tracking and monitoring could be used as a communication tool between the parties in clinical practice. This finding is similar to that of a recent study using participatory design methods, asking patients about their needs, values, and preferences with respect to the use of eHealth tools in the management of their disease [29]. In the latter study, patients advocated the use of such tools to support their preparation for consultations in clinical practice, in order to empower them and make them more active comanagers of their disease.

When implementing a solution, as evaluated in ACQUIRE-HF, in clinical practice, it is important to emphasize that patient tracking and monitoring may induce anxiety in some patients, in particular if they see a significant reduction in their health status. This was mentioned by one of the patients in the post-hoc interviews. On the other hand, several of the patients who were interviewed also mentioned that tracking of their own health status was insightful, constituted a learning opportunity, and for some was an indication of how far they had come in managing their disease. The importance of patients gaining increased awareness about their disease and disease status through eHealth solutions is also supported by others [30]. However, in a recent study in patients with multiple chronic diseases, tracking of objective clinical parameters (eg, self-tracking of blood glucose level, results of blood tests received from the hospital) may not only have emotional but also moral implications, including feelings of guilt in patients who have not been compliant [30,31]. Although we did not explicitly ask patients whether they thought that a reduction in their perceived health status had moral implications and no patients mentioned it in the interviews, it is possible that asking patients to track their own health status is benign, as it provides an overall snapshot of patients’ health [13] rather than specific values, such as blood glucose level and blood pressure. Designing a solution that also focuses on patient strengths and resources, such as optimism and willpower to overcome obstacles, may be paramount to balance the positives and negatives of such solutions at the patient level in future eHealth interventions [29].

For the purpose of the study, we designed a questionnaire to tap into patient expectations toward the platform prior to use and perceived experiences post use. Based on our results, patient expectations toward use of the platform prior to actual use were at a lower level than their actual experiences with the platform at the end of 3 months, suggesting that patients generally experienced gains that they might not have anticipated. In future studies, it will be important to focus on patient expectations toward the eHealth intervention or tools that will be evaluated, as such expectations may not only influence patient engagement and user experience but also patient-reported outcomes [20].

### Limitations

The results of this study should be interpreted with the following limitations in mind. We recruited patients from only one center, and the majority of patients were men. Hence, the results may not necessarily be generalizable to the general HF population and in particular to women. Due to the small sample size, we were not able to perform sophisticated statistical analyses and thus are only able to report simple descriptive statistics. Although the focus of the feasibility study was to evaluate the experiences of patients with HF when tracking and monitoring their own health status and the potential value to nursing staff, we are not able to delineate whether use of the platform had a direct impact on patient HF symptoms, as there could be many alternative reasons as to why patients improved or deteriorated in their health status as measured by the KCCQ.

### Conclusion

The use of patient tracking and monitoring of health status in HF using a standardized and validated measure seems feasible and may lead to insights that will help educate, empower, and engage patients more in their own disease management. Nursing staff found the patient-centered tool beneficial as a communication tool with patients, indicating that the dialog might become more equal and that it might represent a more honest picture of patient cardiovascular health and disease status. However, they were more reticent with respect to using it as a replacement for the personal contact in the outpatient clinic. Further studies are warranted to examine how technology and a more elaborate intervention than provided in our study may facilitate the dialog between health care professionals and patients and improve patient outcomes.

## Abbreviations

ACQUIRE-HF: Advance the Quality of Life and Care of Patients With Heart Failure
CC360: CommunityCare360
EHFScB: European Heart Failure Self-Care Behavior scale
EHR: electronic health record
EXPECT-ICD: Expectations Towards ICD Therapy
HF: heart failure
ICD-10: International Classification of Diseases, 10th Edition
ICD-PC: ICD-10 procedure codes
IT: information technology
KCCQ: Kansas City Cardiomyopathy Questionnaire
NPU: Nomenclature for Properties and Units
NYHA: New York Heart Association
